# Scalable variable-index elasto-optic metamaterials for macroscopic optical components and devices

**DOI:** 10.1038/ncomms16090

**Published:** 2017-07-12

**Authors:** Dongheok Shin, Junhyun Kim, Changwook Kim, Kyuyoung Bae, Seunghwa Baek, Gumin Kang, Yaroslav Urzhumov, David R. Smith, Kyoungsik Kim

**Affiliations:** 1 School of Mechanical Engineering, Yonsei University, 50 Yonsei-ro, Seodaemun-gu, Seoul 03722, Republic of Korea; 2 Centre for Metamaterials and Integrated Plasmonics, Department of Electrical and Computer Engineering, Duke University, Durham, North Carolina 27708, USA

## Abstract

Optical metamaterials with an artificial subwavelength structure offer new approaches to implement advanced optical devices. However, some of the biggest challenges associated with the development of metamaterials in the visible spectrum are the high costs and slow production speeds of the nanofabrication processes. Here, we demonstrate a macroscale (>35 mm) transformation-optics wave bender (293 mm^2^) and Luneburg lens (855 mm^2^) in the broadband white-light visible wavelength range using the concept of elasto-optic metamaterials that combines optics and solid mechanics. Our metamaterials consist of mesoscopically homogeneous chunks of bulk aerogels with superior, broadband optical transparency across the visible spectrum and an adjustable, stress-tuneable refractive index ranging from 1.43 down to nearly the free space index (∼1.074). The experimental results show that broadband light can be controlled and redirected in a volume of >10^5^*λ* × 10^5^*λ* × 10^3^*λ*, which enables natural light to be processed directly by metamaterial-based optical devices without any additional coupling components.

## Abstract (web summary)

Large-scale graded-index metamaterial devices are difficult to fabricate owing to limitations of typical micro- and nanofabrication approaches. Here, Shin *et al*. demonstrate millimetre-scale transformation elements based on elasto-optic metamaterials made from aerogels.

Controlling the propagation of natural visible-spectrum light through macroscopically thick optical components without significant attenuation is a requirement for many advanced optical applications. By introducing a specifically graded refractive index, inhomogeneous photonic materials offer an opportunity for lenses and other broadband optical elements. By designing the artificial subwavelength details of a structure, metamaterials are able to exhibit properties difficult to observe in nature, such as negative or gradient refractive index properties enabling superlenses, optical cloaking or optical elements with gradient indices^1^,^2^,^3^,^4^,^5^,^6^,^7^. Because commonly used nanofabrication techniques, such as electron-beam lithography or focused ion beam patterning, are expensive and time-consuming, optical metamaterials have been demonstrated almost exclusively on microscopic scales, for functions such as light redirection in confined modes of thin planar waveguides or in surface waves^8^. Thus, the typical light-controlling volume (that is, the volume of the region where we can control the fields and distinguishably redirect light rays) has been limited to approximately one thousand cubic wavelengths, or <10*λ* × 10*λ* × 10*λ*. For light to interact with optical devices of volume in the order of wavelength, artificial light couplers are required, such as fibre couplers or grating couplers^9^,^10^. Furthermore, microscopic light manipulation devices typically rely on diffractive phenomena and, thus, are bandwidth-limited. Although ray optics enables devices with multiple-octave bandwidths, it requires much larger volumes of optical components. Macroscopically large, yet mass-producible at a consumer-affordable price, optical metamaterials would therefore provide new opportunities in the photonics markets.

Here, we demonstrate a macroscale (>35 mm) transformation-optics wave bender and a Luneburg lens for natural-light coupling. To combine optics and solid mechanics, we introduce an elasto-optic metamaterial^11^,^12^,^13^,^14^, specifically, a mesoscopically homogeneous aerogel with a nanoporous (∼60 nm) microstructure. Through elastic deformations, we establish and control on-demand graded-index distributions at the meso-to-macro range of scales in a large area or volume. These metamaterials are based on compressible transparent homogeneous aerogels with an ultralow refractive index of 1.074 and a Poisson’s ratio of 0.12. The areas of our two proof-of-concept demonstrators, a wave bender and a Luneburg lens, are 293 and 855 mm^2^, respectively. The vertical thickness of the working devices (∼1 mm) is determined by the maximally compressed region. This shows that wide-spectrum natural-light propagation can be controlled in the metamaterial volume of >10^5^*λ* × 10^5^*λ* × 10^3^*λ* without any extra coupling components.

## Results

### Theory of elasto-optic metamaterials

**Figure 1:**
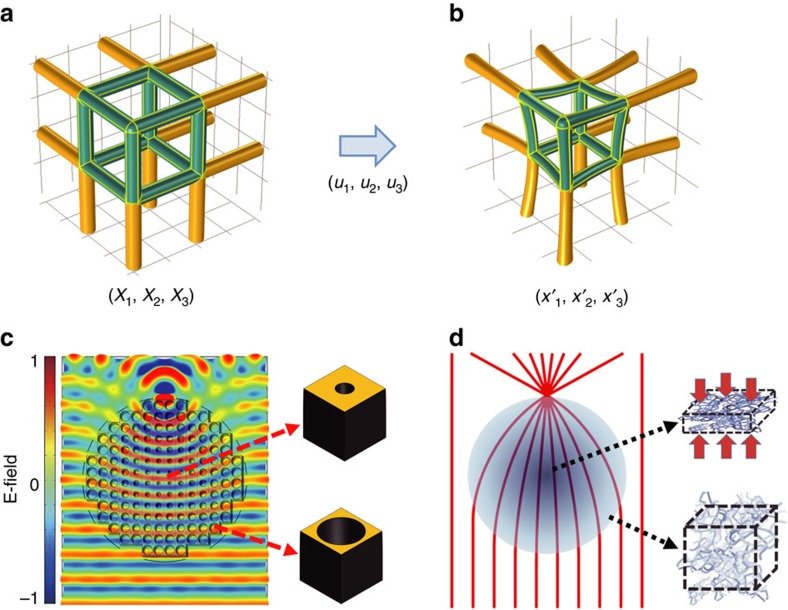
Concept diagrams of an elasto-optic metamaterial and a Luneburg lens. (**a**,**b**) Deformation of the elasto-optic metamaterial. The coordinate transformation from (*X*_1_, *X*_2_, *X*_3_) to (*x*′_1_, *x*′_2_, *x*′_3_) by elastic deformation of displacement (*u*_1_, *u*_2_, *u*_3_) in a network structure of an elasto-optic metamaterial. The green rods in the cube indicate a unit cell of the network structure made of incompressible dielectric materials with an air void. Elastic deformation squeezes the unit cell and the volume of the void, thereby causing changes of the volume fraction between the dielectric and the void and thus the effective index. (**c**) Electric field distribution and a schematic of a Luneburg lens made with conventional metamaterials of the same sized unit cells with different hole geometries. Because of the difficult fabrication process used, the light-controlling volume is <10*λ* × 10*λ* × 10*λ*. (**d**) A schematic of a Luneburg lens made with an elasto-optic metamaterial mechanically deformed from a homogeneous nanoporous material. Using a simple elastic deformation process, the light-controlling volume is >10^5^*λ* × 10^5^*λ* × 10^3^*λ*.

To understand the concept of elasto-optic, mechanically tuneable metamaterials, consider a deformable homogeneous effective medium^15^, such as a binary composite of a stiff dielectric and an air-filled void. There have been some studies that controlled the elastic wave phenomena by use of the solid mechanics concept^16^,^17^,^18^. For simplicity, consider a composite with a periodic arrangement of identical unit cells. An elastic deformation of such a medium changes the refractive index and simultaneously the volume of a representative unit cell, as shown in Fig. 1a,b. Upon deformation, stress and strain fields propagate through the lattice of such cells, and a new mechanical equilibrium is established. Using conventional continuum mechanics, we describe these deformations using a displacement vector field with components (*u*_1_, *u*_2_, *u*_3_), which relates the displacement of a point (*X*_1_, *X*_2_, *X*_3_) to its new position (*x*′_1_, *x*′_2_, *x*′_3_), according to the relationship **X**+**u**=**x**′^19^. We use the deformation gradient tensor 
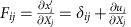
, and express the compression (or expansion) ratio (*J*) through its Jacobian, 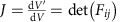. After the deformation, the volume fraction (*f*) of the stiff dielectric component changes from *f*=*V*_*d*_/*V*_*t*_ to *f*′=*V*_*d*_/*V*′_*t*_, resulting in *f*′=(*V*_*d*_/*V*_*t*_)/(*V*′_*t*_/*V*_*t*_)=*f*/*J*, where the volume of the dielectric is *V*_*d*_, and the volume of the total unit cell is *V*_*t*_. By changing the compression ratio (*J*) or volume fraction (*f*), we are able to obtain a wide range of effective refractive indices (*n*_eff_(*J*)) that are predicted in numerical simulations using a retrieval method^20^,^21^,^22^.

Previous demonstrations of metamaterial-based Luneburg lenses were based on periodic or quasi-periodic lattices of fixed-size, variable-hole-geometry metamaterials, as shown in Fig. 1c. Such an approach, while feasible for microwave and even millimetre-wave devices, becomes prohibitively expensive for optical applications. Here, by employing the elasto-optic metamaterial concept, we achieve the required graded-index profiles through a compression ratio distribution. The Luneburg lens can be achieved with a simple elastic deformation process of a homogeneous nanoporous material, as shown in Fig. 1d. On the basis of continuum mechanics, stress and strain are continuously distributed in a deformed network medium, leading to a controllable effective index distribution in elasto-optic metamaterials.

### Aerogels for elasto-optic metamaterials

**Figure 2:**
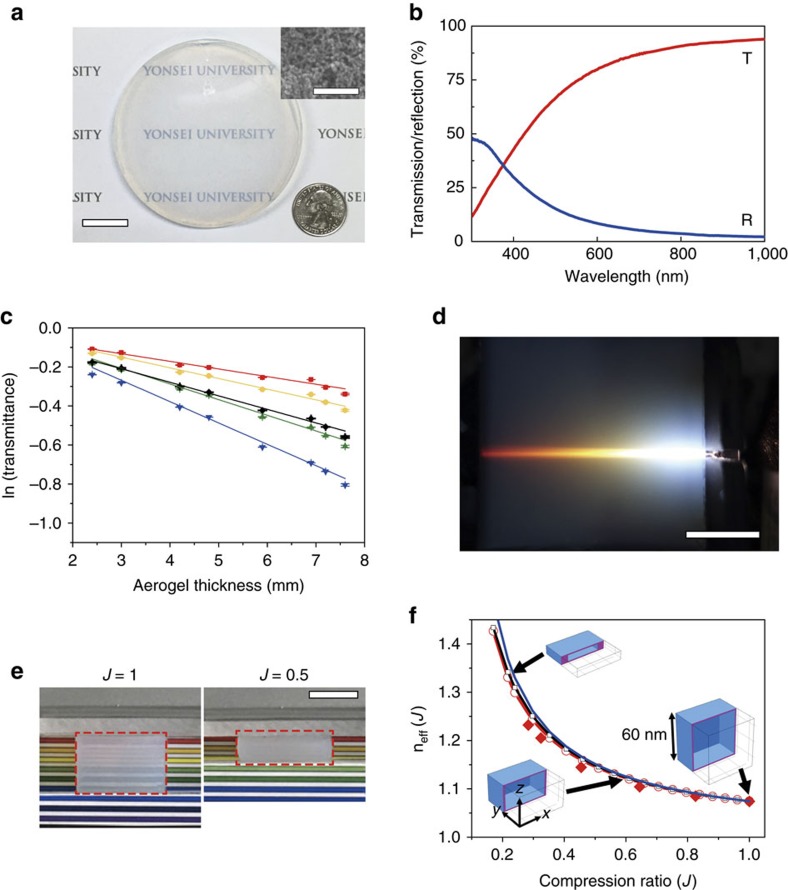
Transparent and compressible aerogel with measured and calculated properties. (**a**) A photograph of a compressible transparent bulk aerogel of 8 cm in diameter, and 1 cm in thickness. The inset shows an SEM image of a nanoporous network structure with 84% porosity. The size of the scale bar, 20 mm (inset 200 nm). (**b**) The measured optical transmittance and reflectance show the good transparency of a 3-mm-thick aerogel in the visible spectrum. (**c**) The measured attenuation coefficients of aerogels at 633 (red), 589 (yellow), 523 (green), 473 (blue) and 400–700 (black) nm are 0.039, 0.055, 0.080, 0.109 and 0.069 mm^−1^, respectively. (**d**) An optical image of white-light propagation in an aerogel. The size of the scale bar, 20 mm. (**e**) Photographs of a compressible aerogel (red dashed box) before (left) and after (right) deformation. The compression ratios (*J*) are 1 and 0.5. The size of the scale bar, 10 mm. (**f**) Using the retrieval method with COMSOL, the calculated effective refractive index (*n*_eff_(*J*)) of the deformed aerogel versus the compression ratios (*J*) for TE (red circle) and TM (black square) polarizations are obtained. The unit cell consists of a dielectric material cubic shell (blue) around an air void with a side length of 60 nm. The dielectric volume fraction is 0.16, the Poisson’s ratio is 0.12, and the undeformed refractive index is 1.074. The simulated shapes of the deformed unit cell are shown for the specific compression ratios of *J*=0.2, 0.6 and 1. *n*_eff_(*J*) from the approximate Clausius-Mossotti (blue line) relation precisely agrees with the retrieval method. The experimentally measured effective indices (red diamond) versus compression ratios of our aerogel chunks agree well with the approximate Clausius-Mossotti relation.

To verify the macroscale elasto-optic concept, differing from conventional rigid and undeformable aerogels, we employ a compressible, transparent aerogel with a nanoporous structure, as presented in Fig. 2a. In our samples, the precursor methyltrimethoxysilane (MTMS) enables us to compress the aerogel in the same manner as a sponge. To reduce the size of granules, we efficiently spread MTMS in the solution using a surfactant, that is, non-ionic surfactant poly(ethylene oxide)-block-poly(propylene oxide)-block-poly(ethylene oxide) triblock copolymer (F127, EO_108_PO_70_EO_108_). Ultrafine nanogranules of ∼20 nm allow us to produce an aerogel that is transparent in the optical regime. The SEM image shows that silica-composite nano-granules form a network structure with ∼60 nm nanopores (Supplementary Fig. 1). The experimentally measured transmittance and reflectance of a 3-mm-thick aerogel show good broadband transparency (Fig. 2b). We measured the attenuation coefficients of aerogels as 0.039, 0.055, 0.080, 0.109 and 0.069 mm^−1^ at 633, 589, 523, 473 and 400∼700 nm, respectively (Fig. 2c), (Supplementary Fig. 2 and Supplementary Table 1). Figure 2d shows the propagation of white light through the aerogel. The aerogel is also compressible by its own spring-back mechanical property with a Poisson’s ratio of 0.12 (Fig. 2e); the deformation process is shown in Supplementary Movie 1. The aerogel can compress from an initial porosity of 84% to nearly zero at the maximum^23^. Finally, we can use the aerogel bulk as an elasto-optic metamaterial in the visible spectrum.

### Optical properties versus compression of aerogels

We experimentally measured the refractive index of our aerogel as *n*_0_=1.078 using Snell’s law^24^ (Supplementary Fig. 3). This ultralow index is close to the index of air because of its high porosity, whereas it can reach as high as *n*∼1.43 when maximally compressed, resulting in a broad tuneable range of Δ*n*∼0.35. The air-like ultralow index of aerogel also minimizes the index-mismatching loss with air at the interfaces. On the basis of the metamaterial retrieval method implemented with a Finite element method (FEM) solver COMSOL, we numerically calculated the effective index (*n*_eff_(*J*)) of the mechanically deformed aerogel versus the compression ratio (*J*)^20^,^21^,^22^. The unit cell comprises a silica cubic shell structure around an air void with a side length of 60 nm (Fig. 2f). The unit cells are elastically deformed in the *z*-direction with a Poisson’s ratio of 0.12, and the light propagates in the *y*-direction with TE or TM polarizations. In a previous study, anisotropic elastic deformation caused negligible birefringence while compressing the aerogels^25^, which is consistent with our simulation results. We approximately simulate the mesoscopically homogeneous aerogels using the Clausius-Mossotti relation, (*n*^2^−1)/(*n*^2^+2)=*Nα*/3, where *N* is the number density of molecules, and *α* is the molecular polarizability. Using *N*=*N*_0_/*J*, after mechanical compression, we can obtain *n*_eff_(*J*) from 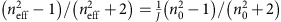, which precisely agrees with the retrieval method. We also experimentally measured the effective indices of our aerogel chunks as a function of the compression ratios; the measurements show good agreement with the approximate model. The measured indices versus various wavelengths are also given in Supplementary Fig. 4 and Supplementary Table 2.

### Design of macroscale elasto-optic metamaterial devices

**Figure 3:**
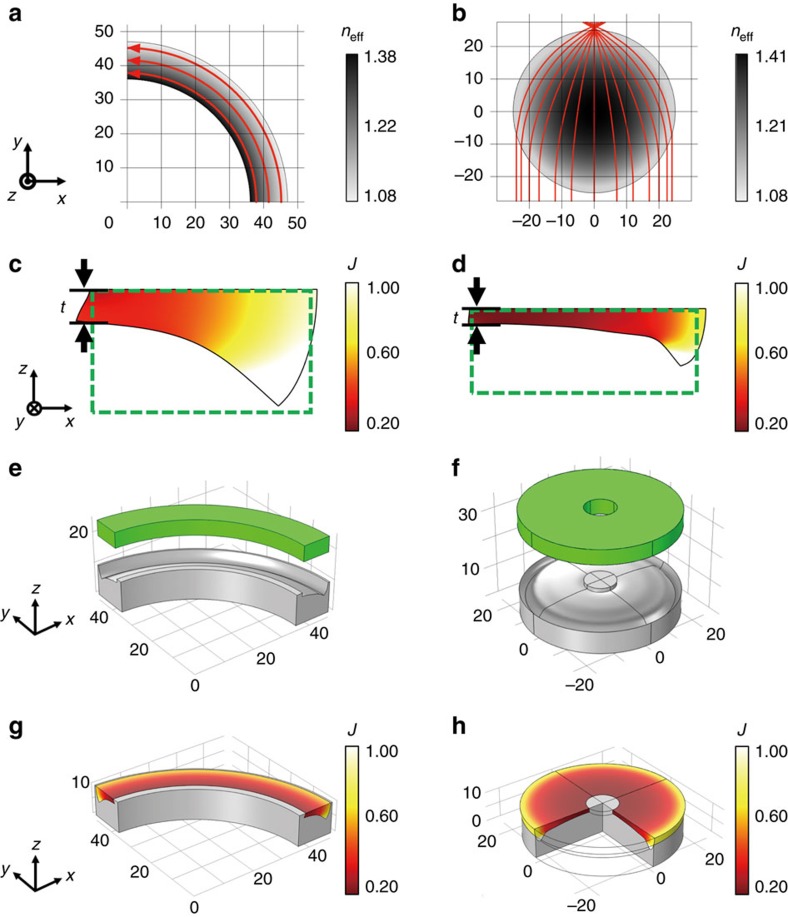
Design and pressing process of a wave bender and a Luneburg lens. (**a**,**b**) COMSOL simulation results of propagating rays on a transformation-optics (TO) wave bender (a) and an optical Luneburg lens (b). 2D refractive-index distributions (grey scale) of a TO wave bender and an optical Luneburg lens are 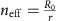 and 
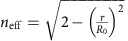
, respectively, where *R*_0_ is the outer radius, and *r* is the distance from the origin in the *xy*-plane. (**c**,**d**) Using the inverse function of the effective index *n*_eff_(*J*) of compressible aerogels, we transformed the designed index distribution into the compression ratio distribution, *J*(*n*_eff_). Dashed lines indicate the cross-sections of the initial homogeneous aerogels. Solid lines show the cross-sectional shapes after compressing to obtain the designed compression ratio and effective index distributions on the top surface. These devices properly operate within the thickness *t*. (**e**,**f**) Designed pressing moulds (grey) and homogeneous aerogel bulks (green) for each device before elastic deformation. (**g**,**h**) Compression profiles in the TO wave bender and the optical Luneburg lens achieved by compressing homogeneous aerogels with pressing moulds in the *z*-direction. Appropriately deformed aerogel bulks have the desired compression ratio and index distributions. Thermal colour bars indicate the compression ratio (**c**,**d**,**g**,**h**).

Next, we produced macroscale natural-light-controlling two-dimensional (2D) metamaterial devices in the *xy*-plane by compressing the aerogel in the out-of-plane *z*-direction. The two examples of graded-index devices selected were: a transformation-optics (TO) wave bender and a Luneburg lens. In the TO wave bender, incident light can propagate in an arbitrary path defined by the shape of the bender while maintaining its initial phase information^26^,^27^,^28^, somewhat similar to wave propagation through an optical fibre. The Luneburg lens has a graded-index distribution such that it focuses plane waves from all directions to a focal point without aberrations^29^,^30^. To date, there have been only microscale demonstrations of optical Luneburg lenses because of the limitations of current nanofabrication technology^31^,^32^,^33^,^34^. Here, an arc-shaped TO wave bender and a Luneburg lens are designed as *n*_eff_=*R*_0_/*r* and 
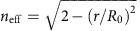
, respectively^26^,^29^, where *R*_0_ is the outer radius, and *r* is the distance from the origin (Fig. 3a,b). From the desired graded indices of the target objects, we obtain the 2D compression ratio (*J*) distribution for the aerogel using the inverse function of *n*_eff_(*J*), calculated in Fig. 2f. To obtain the designed compression ratio on the *xy*-plane, we calculate the cross-sectional shape resulting from a deformation in the *z*-direction using the solid mechanics module in COMSOL Multiphysics (Fig. 3c,d, Supplementary Fig. 5). As we deform homogeneous rectangular aerogel chunks (dashed lines) into solid line shapes by compressing the moulds, we obtain the desired index distribution on the top surface region. These devices are operating as described for light propagating within the cross-sectional thickness (*t*) determined by the maximally compressed region, where the distributions of compression ratio (*J*) are almost constant along the *z*-direction. By rotating the cross-sections, we designed the three-dimensional (3D) pressing moulds and fabricated with a 3D printer (3Dwox, Sindoh) (Fig. 3e,f). The initial undeformed homogeneous aerogels are presented as green structures. For the Luneburg lens, we use an aerogel plate with a hole to reduce the resistive force for compression in the centre region. After we deform the aerogels by pressing the moulds, we obtain the required compression ratio distributions for the wave bender and the Luneburg lens (Fig. 3g,h).

### Demonstrations of macroscopic optical devices

**Figure 4:**
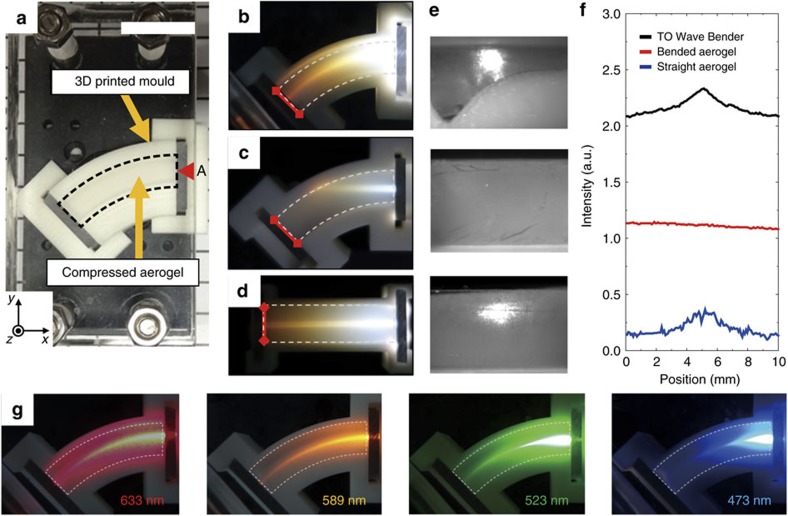
Experimental demonstrations of a macroscale visible wave bender. (**a**) A photograph of the wave bender sample. The size of the scale bar, 20 mm. With a 3D printed pressing mould for wave bender, we compressed the aerogel (dashed region), resulting in the designed distribution of the compression ratio and the effective refractive index for the transformation-optics (TO) wave bender. The aerogel prepared for the wave bender sample with 45° bending has the following parameters: outer radius *R*_0_=46 mm, inner radius *r*_0_=37 mm, and thickness *t*_0_=5 mm. The light beams are incident on the red spot A from the right. (**b**) A collimated white-light beam from a solar simulator is incident and propagates along the curved wave bender, satisfying the TO design. For reference, light propagates in a straight line inside (**c**) a 45°-bend, and (**d**) a straight homogeneous aerogel chunk, without mechanical deformation. (**e**) The cross-sectional images and (**f**) intensity profiles of light beams at the output surfaces (red edges in **b**,**c**,**d**. (**g**) Various wavelength laser beams (633, 589, 523 and 473 nm) are incident and propagate along the curved wave bender, satisfying the TO design.

To demonstrate the practical feasibility of our approach, we prepared the described macroscale optical devices by mechanically deforming homogeneous aerogels. We fabricated a homogeneous arc-shaped aerogel bulk for the wave bender with a 45° bend (outer radius *R*_0_=46 mm, inner radius *r*_0_=37 mm, thickness *t*_0_=5 mm) and an aerogel plate with a central hole for the Luneburg lens (outer radius *R*_0_=16.5 mm, inner radius *r*_0_=2.5 mm, thickness *t*_0_=5 mm). After sandwiching the homogeneous aerogel bulk with 3D printed pressing moulds and acryl top windows, we elastically compressed the aerogel on an aluminium plate into the designed shapes of Fig. 3g,h, leading to the index distributions of the target devices within the 1-mm-thick top surface region. Figure 4a presents a TO wave bender realized by elasto-optic metamaterials after mechanical compression. To investigate the TO wave-guiding characteristics, as references, we also prepared a 45°-bend aerogel chunk and a straight homogeneous aerogel chunk without mechanical deformation. When a collimated white-light beam from a solar simulator (YCSS-50, Yamashita Denso) is incident on spot A from the right side, the broadband light propagates along the curved path, as designed by the TO (Fig. 4b). Figure 4c,d describe the straight light propagations in homogeneous aerogel chunks. At the output surfaces of samples (red edges in Fig. 4b–d), the cross-sectional images (Fig. 4e) and intensity profiles (Fig. 4f) of the light beams clearly show the TO wave-bending characteristics of the mechanically compressed elasto-optic metamaterial. The light intensity decays because nanopores in the aerogel scatter the propagating light; the average attenuation coefficient for a broad range of wavelengths from 400 to 700 nm is 0.069 mm^−1^. The attenuation constants at various wavelengths are also given in Supplementary Table 1. In the TO wave bender, laser beams also propagate along the same curved path at various wavelengths of 633, 589, 523 and 473 nm (Fig. 4g). We used low-power cw laser sources to eliminate any nonlinear effects, such as thermal lensing^35^.

**Figure 5:**
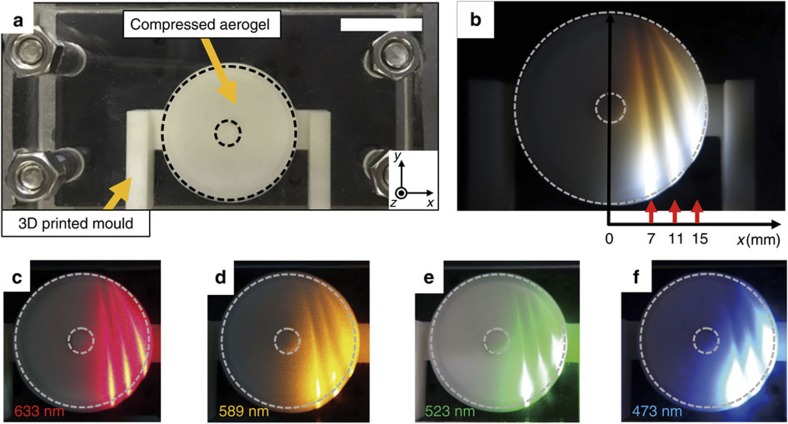
Experimental demonstrations of a macroscale visible Luneburg lens. (**a**) A photograph of the Luneburg lens sample (33 mm diameter) with a central hole (5 mm diameter). The size of the scale bar, 20 mm. Using the 3D printed pressing mould for Luneburg lens, we compressed the aerogel (dashed region), resulting in the designed distribution of compression ratio and effective refractive-index for the Luneburg lens in Fig. 3h. The aerogel prepared for the Luneburg lens sample has the following parameters: outer radius *R*_0_=16.5 mm, inner radius *r*_0_=2.5 mm and thickness *t*_0_=5 mm. (**b**) White-light beams (red arrows) are incident in the *y*-direction at varying input positions in the *x*-direction (7, 11, 15 mm). The light beam is redirected into a broad focal position at the output surface. (**c**–**f**) Various wavelength laser beams (633, 589, 523 and 473 nm) are incident and redirected into a similar broad focal position on the output surface.

Figure 5a shows a macroscale visible Luneburg lens of 3.3 cm in diameter made by a deformed aerogel. When white-light beams are incident in the *y*-direction, as we change the *x*-positions for incidence, the Luneburg lens has a clear tendency to redirect the broadband light into a broad focal position at the output surface (Fig. 5b). Because the focusing performance is limited by the shear modulus properties of aerogels, further research is required to realize an ideal Luneburg lens. The lens shows similar focusing behaviour for laser beams at the wavelengths of 633, 589, 523 and 473 nm (Fig. 5c–f). For a larger Luneburg lens of 5 cm in diameter, the same light-redirecting tendency was demonstrated (Supplementary Fig. 6).

## Discussion

These results demonstrate the feasibility of large-area broadband natural-light optical devices based on elasto-optic metamaterials. The desired gradient-index profiles are achieved without nanofabrication by virtue of stressing our reversibly compressible transparent homogeneous aerogels. In the Luneburg lens demonstration, the total optical path that can be controlled within the device can be as long as ∼50 mm, that is ∼10^5^*λ*. The cross-sectional areas of the wave bender and the Luneburg lens are 293 and 855 mm^2^, respectively, with the thickness of the functional layer being approximately 1 mm.

Elasto-optic metamaterials enable the development of optical devices with a very large lateral area, transverse thickness and volume that exceed previous optical metamaterial device dimensions by many orders of magnitude. This technique enables precise control over broadband light propagation in volumes as large as 10^13^*λ*^3^ (for example, 10^5^*λ* × 10^5^*λ* × 10^3^*λ*). This allows natural light to interact directly with metamaterial devices without any additional coupling components, opening the door to industrial applications of optical metamaterials in general, and transformation optics in particular, for example, adaptive lenses for advanced miniaturized cameras, machine vision, lidar-based technologies and energy harvesting.

## Methods

### Deformation gradient tensor in solid mechanics

If we consider an elastic deformation process, there are two explicit coordinates, that is, the original flat space **X** and the mechanically deformed space **x**′=**X**+**u**, where the vector field is the displacement field **u**, as described in Supplementary Fig. 7. To quantify the change in the shape of a deformed solid body, the concept of the deformation gradient tensor is introduced as 
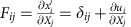
. The Jacobian (*J*) of the deformation gradient is defined as 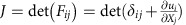 and represents the ratio of the deformed volume over the initial volume according to the relationship 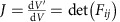. For an incompressible solid, *J*=1. If *J*>1 (*J*<1), the solid expands (is compressed).

### Fabrication of the aerogel

For the fabrication of the aerogel, we prepared the sol by mixing solutions of 10 mM aqueous acetic acid, MTMS, urea and the surfactant (F127) with the mass ratios of 7:4.76:0.5:1.1 and then thermally heated the sol for gelation in an oven at 60 °C for 4 days. Next, we soaked the obtained wet-gel in 2-propanol at 60 °C for 3 days with two solvent exchanges. Finally, we dried the wet-gel using a supercritical drying process. The completely dried nanoporous aerogel consists of 16% polymethylsilsesquioxane (PMSQ) network and 84% voids^36^.

### Measurement of the compressed aerogel refractive index using Snell’s law

Following the method from ref. 24, we experimentally measured the refractive index of a compressed aerogel as shown in Supplementary Fig. 3. If an incident beam entered a rectangular aerogel at an oblique angle (45°, 50°, 55°, 60° in our set-up), the two refractions at the input and output surfaces of the aerogel exhibit a large lateral shift, *δ*_*y*_, for the laser beam propagation. By measuring the values of *δ*_*x*_, *δ*_*y*_, *θ*_1_ and *β* in the experiment, we obtain *θ*_2_, *θ*_3_, and the refractive index of aerogels using Snell’s law. To characterize the effective index of a compressed aerogel, we experimentally measured the effective indices of an aerogel chunk at different compression ratios. Supplementary Fig. 4 presents the effective refractive indices of a compressed aerogel measured by laser beams of different wavelengths (633, 589, 523 and 473 nm) for the compression ratios of 0.28, 0.32, 0.45, 0.64, 0.83 and 1. The measured values for 633 nm are presented with the theoretical results in Fig. 2f of the manuscript. The experimental results are summarized in Supplementary Table 2.

### Optical measurement of transmittance and reflectance of the aerogels

To measure the transmittance and reflectance of the aerogels in the wavelength range of 300–1,000 nm, we used a ultraviolet–visble–NIR spectrometer (UV3600, Shimadzu Scientific Instruments) with a 60-mm-diameter integrating sphere (MPC-3100) by scanning a monochromator coupled to a halogen lamp. The transmitted and reflected light from the aerogels was scattered and collected in an integrating sphere and then detected with a photomultiplier tube. Using an integrating sphere, we can thoroughly measure the total reflectance *R*(*λ*) (transmittance (*T*(*λ*)) spectra of aerogels with scattering from ∼60 nm nanopores, including both diffuse and specular reflections (transmissions). For the measurement of the reflection/transmission spectra, we mounted the samples at an oblique incidence angle (8°) with respect to the normal at the rear/front of an integrating sphere.

### Measurement of the attenuation coefficient of the aerogels

To characterize the attenuation coefficient of the aerogels, we measured the total spectral transmittance of aerogels with various thicknesses as described in Supplementary Fig. 2. We used a ultraviolet–visble–NIR spectrometer (UV3600, Shimadzu Scientific Instruments) with a 60-mm-diameter integrating sphere (MPC-3100). Supplementary Fig. 2a presents the transmittance of the aerogels in the wavelength range of 400–850 nm with thicknesses of 2.4, 3.0, 4.2, 4.8, 5.9, 6.9, 7.2 and 7.6 mm. The transmittance reduction during light propagation is lower at longer wavelengths. This results in a smaller attenuation coefficient at longer wavelengths; consequently, red light can propagate for longer distances in the aerogels compared with light at shorter wavelengths (Supplementary Fig. 2b). Figure 2c shows the attenuation in the aerogels as a function of the propagation length (the thickness of aerogels) on a semi-logarithmic scale at wavelengths of 633, 589, 523 and 473 nm. The attenuation for an averaged transmittance in the visible spectrum from 400 to 700 nm is also plotted. The slope of the fit gives an estimate of the attenuation coefficient (in mm^−1^) of each wavelength; the results are summarized in Supplementary Table 1.

### Calculation of the optical properties versus the compression of aerogels

To calculate the optical properties as the aerogels are mechanically compressed, it is necessary to find the parameter relating the elasticity and the optical properties in the same material. Using the solid mechanics and optics modules of COMSOL simulation, we find the relationship between the strain tensor (induced by elastic deformation) and the permittivity and permeability tensors. Because the optical birefringence generated by anisotropic elastic compression is reported to be negligibly small^25^, the permeability and permittivity tensors can be represented with the isotropic refractive index. The strain tensor can also be quantified using the volume compression ratio (*J*). We modelled a unit cell of the elasto-optic metamaterial with a single pore surrounded by a dielectric shell. As the unit cell is mechanically compressed, we can obtain the refractive index versus the compression ratio (*J*) using the metamaterial retrieval method^20^,^21^,^22^. The results are shown in Fig. 2f.

### Data availability

The authors declare that the data supporting the findings of this study are available within the paper and its Supplementary Information files.

## Additional information

**How to cite this article:** Shin, D. *et al*. Scalable variable-index elasto-optic metamaterials for macroscopic optical components and devices. *Nat. Commun.* **8**, 16090 doi: 10.1038/ncomms16090 (2017).

**Publisher’s note:** Springer Nature remains neutral with regard to jurisdictional claims in published maps and institutional affiliations.

## Supplementary Material

Supplementary Information

Supplementary Movie 1

Supplementary Movie 2
