# Supplementary material for: Scalable variable-index elasto-optic metamaterials for macroscopic optical components and devices
Source: Nat Commun. 2017 Jul 12;8:16090. doi: 10.1038/ncomms16090 (PMC5510221; doi:10.1038/ncomms16090)
Supplement: Supplementary Information [file ncomms16090-s1.pdf]

File name: Supplementary Information
Description: Supplementary Figures, Supplementary Tables and Supplementary References

File name: Supplementary Movie 1
Description: The movie shows compression behaviour of the aerogel in Fig. 2d of the manuscript.

File name: Supplementary Movie 2
Description: The movie shows that a coloured laser beam (633 nm) is incident and redirected into a broad focal position on the output surface of the Luneburg lens in Fig. 5c of the manuscript.

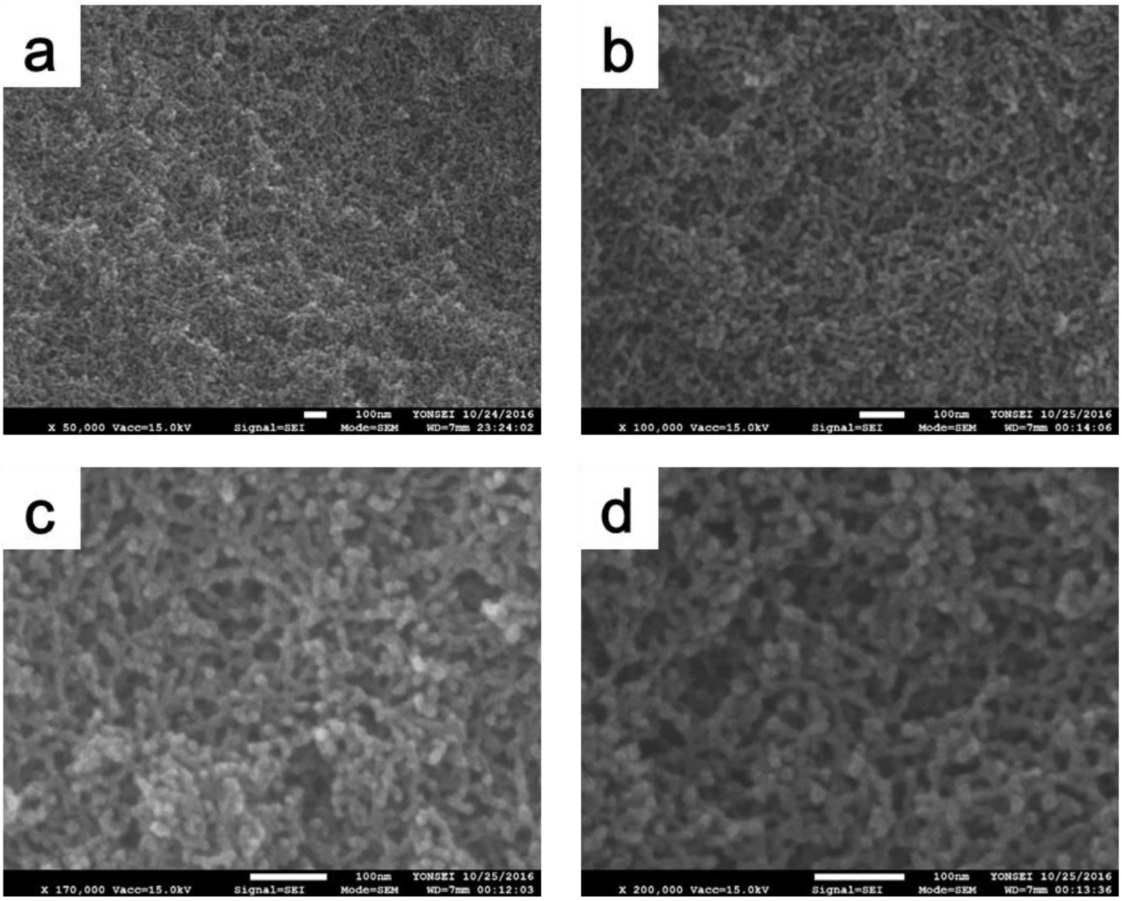

Supplementary Figure 1 Scanning electron microscope image of aerogel surfaces under different magnifications.

The scanning electron microscope images of aerogel surfaces with magnification of **a,** 50,000x, **b,** 100,000x, **c,** 170,000x and **d,** 200,000x respectively. Each scale bar indicates 100 nm.

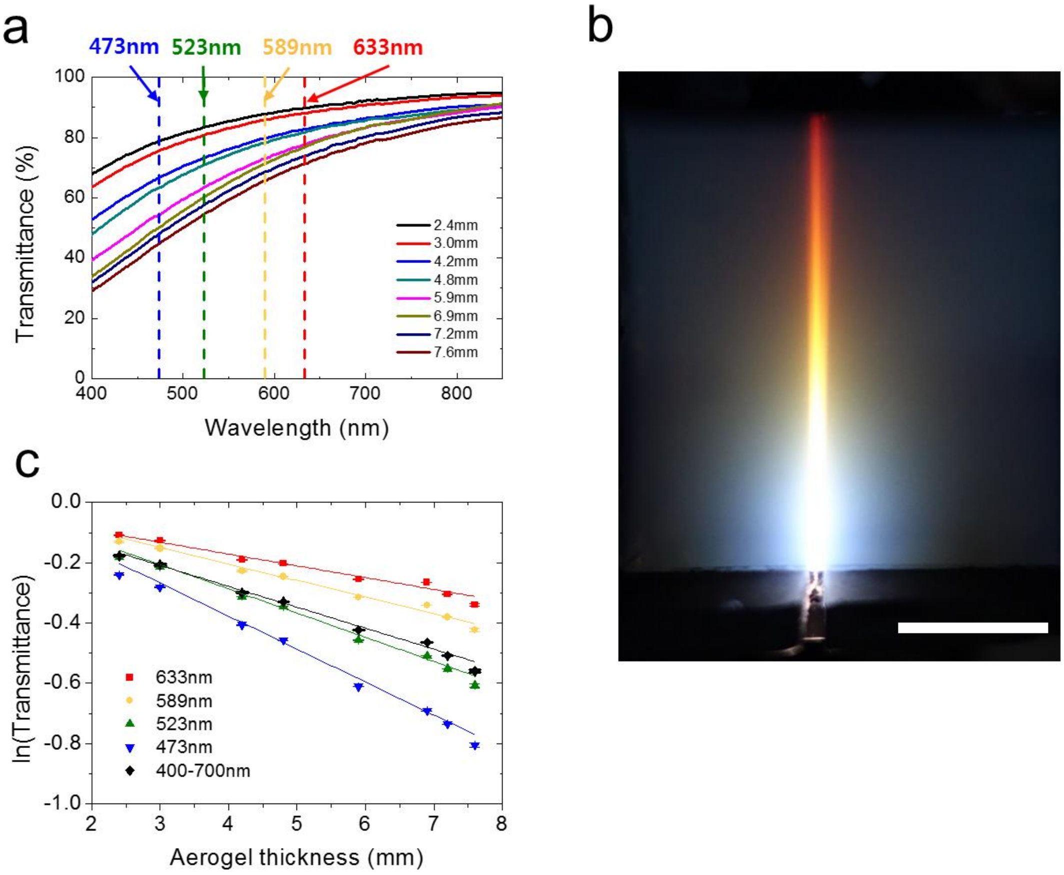

Supplementary Figure 2 Measurement of attenuation coefficient of aerogels.

a, The measured optical transmittance of aerogels with various thicknesses. The dashed lines are wavelengths of lasers which were used to observe the propagation of light. **b,** An optical image of white light propagation in an aerogel. The size of the scale bar is 20 mm **c,** Fit of the attenuation data of aerogels at the wavelengths of 633 (red), 589 (yellow), 523 (green) and 473 nm (blue). The black solid line is a fit for an averaged transmittance in the visible range (400 – 700 nm).

Supplementary Table 1 Attenuation coefficients of aerogels

Wavelength (nm)	Attenuation coefficient (mm^{-1})
633	0.039 ±0.003
589	0.055 ±0.002
523	0.080 ±0.003
473	0.109 ±0.005
400 – 700	0.069 ±0.004

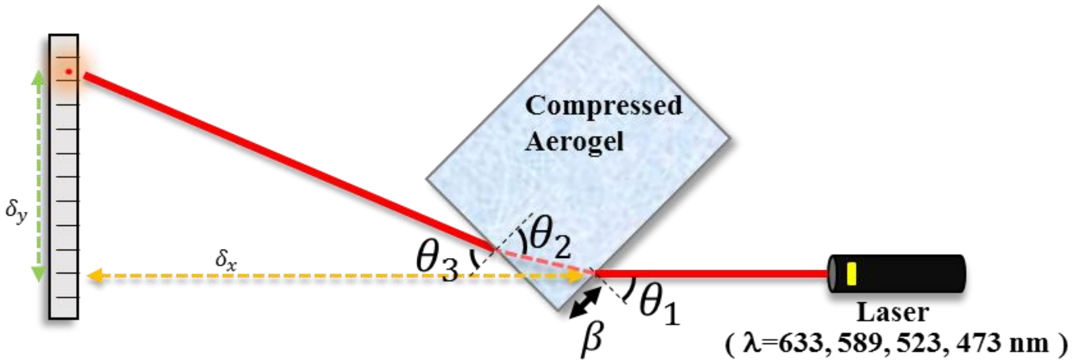

Supplementary Figure 3 A diagram of experimental setup for the index measurement of compressed aerogel.

To experimentally measure the refractive index of compressed aerogels, we illuminate a laser beam of various wavelengths (633, 589, 523, 473 nm) into a rectangular aerogel sample at an oblique angle θ_1. The incident laser beam refracts at input and output surfaces of the aerogel, thus make a large lateral shift, δ_y, as shown in the figure. By measuring the values of δ_x, δ_y, θ_1 and β in the experiment, we get θ_2, θ_3, and the refractive index of the compressed aerogel sample using Snell's law[1].

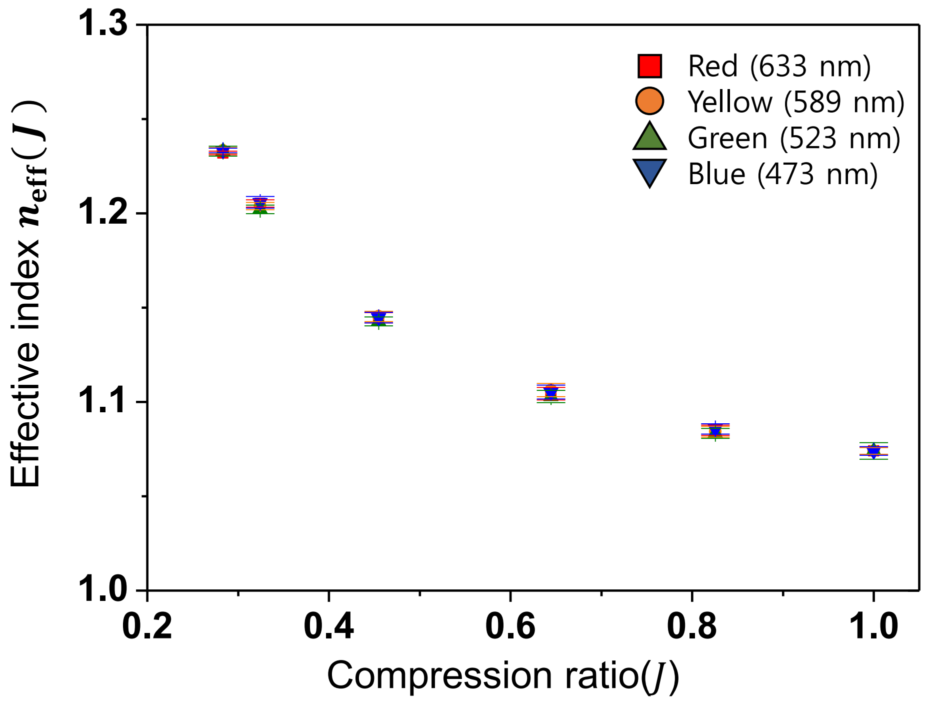

Supplementary Figure 4 The measured effective index of refraction of compressed aerogels for various compression ratios.

We measured the effective indices of compressed aerogels [1] at various wavelengths (633, 589, 523, 473 nm) with the compression ratios of 0.28, 0.32, 0.45, 0.64, 0.83 and 1. While we compress the aerogel samples, to confirm the validity of our measurement, we used varying parameters for distance δ_x (250, 300, 350, 400, 450 mm) and the incidence angle θ_1 (45°, 50°, 55°, 60°). We used five aerogel samples and measured the refractive indices while compressing. After we got data for different parameters of δ_x (250, 300, 350, 400, 450 mm) and θ_1 (45°, 50°, 55°, 60°), error bars are obtained from their standard deviations.

Supplementary Table 2 Measured effective indices of the compressed aerogel.

For the refractive indices with different compression ratio (J) and wavelengths (633, 589, 523, 473 nm), we experimentally measured [1] for the cases of different distances δ_x (250, 300, 350, 400, 450 mm) and incidence angle θ_1 (45°, 50°, 55°, 60°). Then the average values and the standard deviations are summarized in this table.

Comp. ratio(J)	Red (633 nm)		Yellow (589 nm)		Green (523 nm)		Blue (473 nm)	
	Average	Std. dev.	Average	Std. dev.	Average	Std. dev.	Average	Std. dev.
1.00	1.074	0.0021	1.074	0.0018	1.074	0.0044	1.074	0.0023
0.83	1.085	0.0027	1.084	0.0028	1.083	0.0026	1.086	0.0027
0.64	1.104	0.0033	1.106	0.0036	1.103	0.0033	1.105	0.0037
0.45	1.145	0.0027	1.145	0.0028	1.143	0.0024	1.145	0.0028
0.32	1.205	0.0019	1.204	0.0019	1.202	0.0022	1.206	0.0030
0.28	1.232	0.0010	1.233	0.0015	1.233	0.0026	1.233	0.0012

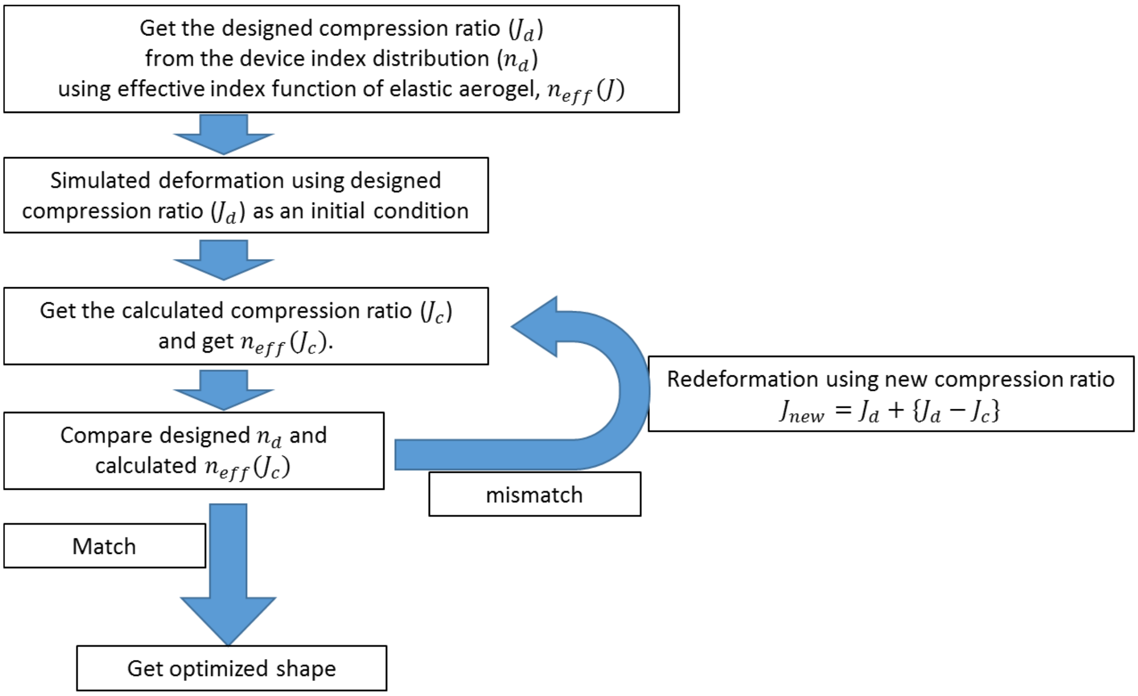

Supplementary Figure 5 A schematic of the algorithm to find out the cross-sectional shape of the deformed aerogels.

At first, we calculate the index profiles of TO or other gradient index device such as TO wave bender or Luneburg lens. The 2D index profiles in this manuscript have axial symmetries, so we can transform the 2D index profiles into 1D index profiles. From the 1D index profile we can achieve compression ratio (J_d) using effective index function of elastic aerogel ($n_{eff}(J)$).

Now we can assume our structure has axial symmetry and a 1D index (compression ratio) profile on the top surface region. To achieve such profile, the structure must be reconstructed by deforming from an initially stress-strain-free bulk medium (undeformed rectangular solid). In COMSOL, we can calculate the deformed structure satisfying the compression ratio on the top surface region. Because the compression ratio reconstructed by COMSOL simulation is a determinant of complex strain and stress tensors, it is hard to achieve the deformed shape with only scalar compression ratio profile. Therefore we calculate the deformed structure with compression ratio profile as an initial strain in axial direction, and recalculate with slightly corrected conditions until we get similar compression ratio profile compare to reference one. If

we get the matched compression ratio, the resulting shape of deformed solid is the gradient optical device with the index profile we designed.

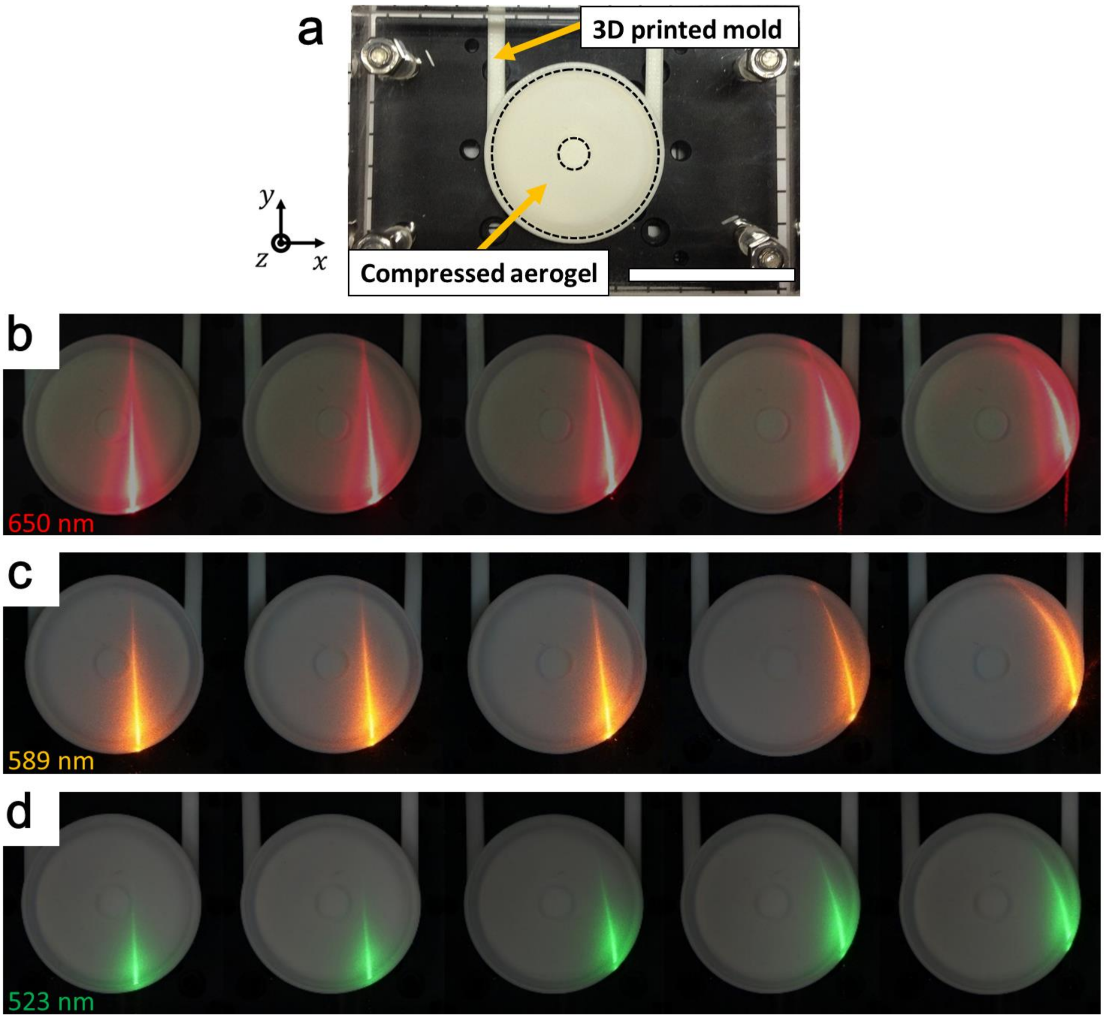

Supplementary Figure 6 A Luneburg lens with a diameter of 5 cm

a, A photograph of the Luneburg lens sample (50 mm diameter) with a centre hole (10 mm diameter). The size of the scale bar is 20 mm. With a 3D printed pressing mold, we compress aerogel (dashed region), resulting in the designed distribution of compression ratio and effective refractive index for Luneburg lens. The aerogel prepared for the Luneburg lens sample has outer radius $R_0 = 25$ mm, inner radius $r_0 = 5$ mm, and thickness $t_0 = 5$ mm. **b-d,** Various wavelength laser beams of (b) 633, (c) 589 and (d) 523 nm are incident in *y*-direction at varying input positions in *x*-direction. The lights are redirecting into a broad focal position at output surface.

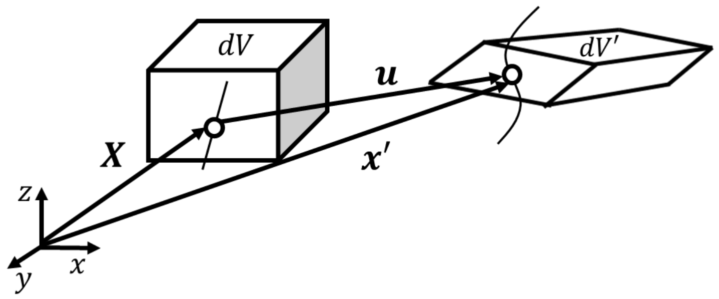

Supplementary Figure 7 A schematic of a solid deformation.

In elastic deformation process, there are two explicit coordinates before and after the deformation: the original flat space $\mathbf{X}$ and the elastically deformed space $\mathbf{x}' = \mathbf{X} + \mathbf{u}$, where the vector $\mathbf{u}$ is the displacement field. The deformation gradient tensor is introduced as $F_{ij} = \frac{\partial x'_i}{\partial X_j} = \delta_{ij} + \frac{\partial u_i}{\partial X_j}$ to quantify the shape change of **a** deformed solid body. The Jacobian (J) of the deformation gradient tensor represents the ratio of deformed volume (dV') over initial volume (dV), which satisfies $J = \frac{dV'}{dV} = \det(F_{ij}) = \det\left(\delta_{ij} + \frac{\partial u_i}{\partial X_j}\right)$. For incompressible solid, for example, J=1. If J>1 (J<1), the solid expands (is compressed).

Supplementary References:

1. Buzykaev, A. R., Danilyuk, A. F., Ganzhur, S. F., Kravchenko, E. A., & Onuchin, A. P. Measurement of optical parameters of aerogel. *Nucl. Instr. Meth. Phys. Res.* A **433**, 396-400 (1999).
